# Pharmacogenomics of COVID-19 therapies

**DOI:** 10.1038/s41525-020-00143-y

**Published:** 2020-08-18

**Authors:** Takuto Takahashi, Jasmine A. Luzum, Melanie R. Nicol, Pamala A. Jacobson

**Affiliations:** 1 Department of Experimental and Clinical Pharmacology, College of Pharmacy University of Minnesota, Minneapolis, MN USA; 2 Division of Hematology/Oncology/Blood and Marrow Transplantation, Department of Pediatrics, University of Minnesota, Minneapolis, MN USA; 3 Department of Clinical Pharmacy, University of Michigan College of Pharmacy, Ann Arbor, MI USA

**Keywords:** Genetic markers, Viral infection

## Abstract

A new global pandemic of coronavirus disease 2019 (COVID-19) has resulted in high mortality and morbidity. Currently numerous drugs are under expedited investigations without well-established safety or efficacy data. Pharmacogenomics may allow individualization of these drugs thereby improving efficacy and safety. In this review, we summarized the pharmacogenomic literature available for COVID-19 drug therapies including hydroxychloroquine, chloroquine, azithromycin, remdesivir, favipiravir, ribavirin, lopinavir/ritonavir, darunavir/cobicistat, interferon beta-1b, tocilizumab, ruxolitinib, baricitinib, and corticosteroids. We searched PubMed, reviewed the Pharmacogenomics Knowledgebase (PharmGKB^®^) website, Clinical Pharmacogenetics Implementation Consortium (CPIC) guidelines, the U.S. Food and Drug Administration (FDA) pharmacogenomics information in the product labeling, and the FDA pharmacogenomics association table. We found several drug-gene variant pairs that may alter the pharmacokinetics of hydroxychloroquine/chloroquine (CYP2C8, CYP2D6, SLCO1A2, and SLCO1B1); azithromycin (ABCB1); ribavirin (SLC29A1, SLC28A2, and SLC28A3); and lopinavir/ritonavir (SLCO1B1, ABCC2, CYP3A). We also identified other variants, that are associated with adverse effects, most notable in hydroxychloroquine/chloroquine (G6PD; hemolysis), ribavirin (ITPA; hemolysis), and interferon β -1b (IRF6; liver toxicity). We also describe the complexity of the risk for QT prolongation in this setting because of additive effects of combining more than one QT-prolonging drug (i.e., hydroxychloroquine/chloroquine and azithromycin), increased concentrations of the drugs due to genetic variants, along with the risk of also combining therapy with potent inhibitors. In conclusion, although direct evidence in COVID-19 patients is lacking, we identified potential actionable genetic markers in COVID-19 therapies. Clinical studies in COVID-19 patients are deemed warranted to assess potential roles of these markers.

## Introduction

Since the report of the first cluster infections in Wuhan, a city in China in December 2019, Coronavirus disease 2019 (COVID-19) has caused an unprecedented global pandemic and healthcare crisis with high mortality and morbidity. In an urgent attempt to mitigate its devastating catastrophe, many drugs without established efficacy have been used in patients either as an off-label/compassionate use or as a clinical trial. Under these extenuating circumstances these agents have been used without good evidence of efficacy and/or extent of toxicities. There is also no or limited data on the pharmacogenomics of these agents, and genomic determinants are important factors in efficacy and/or toxicity of many medications. Pharmacogenomics may help clinicians to choose proper first-line agents and initial dosing that would be most likely achieve adequate drug exposure among critically ill patients; those who cannot afford a failure of ineffective therapy. It is also important to minimize the risks of toxicity because COVID-19 particularly affects those with comorbidities on other drug therapies^1^. Therefore the purpose of this review was to summarize the pharmacogenomic literature and clinical recommendations available for COVID-19 candidate drug therapies.

## Methods

In selection of drugs of interest, we reviewed the following sources: guidelines by the Infectious Disease Society of America^2^ and the National Institute of Health^3^. We also reviewed interventional clinical trials for COVID-19 in ClinicalTrials.gov registered as of June 4, 2020 (searched by a term “COVID” and selected the study type “interventional [clinical trials]”). We excluded products derived of human plasma (e.g., convalescent plasma, immunoglobulin). Our search was limited to online sources published in English. As a result, we included potential antiviral or immune-based therapy in the following eight categories for the present review: hydroxychloroquine/chloroquine, azithromycin, RNA polymerase inhibitors, anti-retrovirus agents, interferon β-1b (IFN-β1b), IL-6/IL-1 antagonists, Janus kinase inhibitors, and corticosteroids. We then reviewed relevant literature by searching PubMed with the terms including but not limited to “pharmacogenomics”, “pharmacogenetics”, “polymorphism”, and “pharmacokinetics” in combination with each drug name. We also reviewed the Pharmacogenomics Knowledgebase (PharmGKB^®^) website^4^, Clinical Pharmacogenetics Implementation Consortium (CPIC) guidelines^5^, the U.S. Food and Drug Administration (FDA) pharmacogenomics information in the product labeling^6^, and the FDA pharmacogenomics association table^7^. None of the reviewed drugs had applicable CPIC guidelines. Table 1 summarizes the drugs described in this review and the PharmGKB and FDA pharmacogenomic information.

**Table 1:** Pharmacogenomics review on potential COVID-19 antivirals and immune-based therapy.

Drug	ClinicalTrials.gov (# of trials as of 6/4/2020)^a^	FDA label/PGx^b^	PharmGKB: Clinical annotations (Level of evidence: Effects)c	Other reported genes	Metabolism/transporter	Adverse effects
Hydroxychloroquine, chloroquine	175	*G6PD/−*	*G6PD* (3: Hemolysis)	1) *ABCA4* (Retinopathy)^19^ 2) *CYP2D6* (Metabolism of HCQ)^15^ 3) *CYP2C8, SLCO1A2, SLCO1B1* (Efficacy in malaria)^14,16^	Metabolism via CYP2C8, CYP3A4, CYP2D6. Influx cellular transport via SLCO.	Hemolytic anemia, cardiomyopathy, neutropenia, GI disturbances, retinopathy, rash, QT prolongation
Azithromycin	60	−/−	*ABCB1* (N/A^d^: PK)	–	Efflux transport via P-glycoprotein.	QT prolongation, CV events
Remdesivir	12	Not approved in US	–	–	Potential inactivation via CYP2C8, CYP2D6, and CYP3A4	None noted (similar to placebo in severe COVID patients)
Favipiravir	19	Not approved in US	–	–	Inactivation mainly via aldehyde oxidase and partly via xanthine oxidase.	Increased uric acid
Ribavirin	2	−/−	*ITPA* (2B: Hemolysis, thrombocytopenia)	1) *SLC28A2, SLC28A3, SLC2A1* (PK)^69^	Intestinal uptake via CNTs (encoded by *SLC*).	Hemolytic anemia
Lopinavir/ritonavir	34	−/−	*ABCB1, ABCC1* (3: Efficacy in HIV); *ABCC2, SLCO1B* (4: PK)	1) *CYP3A4, CYP3A5, SLCO1B1, ABCC2, DRD3, MDR1* (PK)^39,70,71^ 2) *CETP, MCP1, ABCC2, LEP, SLCO1B3, IL6* (Toxicity)^40^	Inactivation via CYP3A4	QT prolongation, CV events, dyslipidemia, liver injury, GI disturbances
Darunavir/cobicistat	1	−/−	*SLCO3A1* (N/A^d^: PK)	–	Inactivation via CYP3A4	Liver injury, dyslipidemia, sulfonamide allergy
Interferon beta-1b	4	−/−	*IRF6* (3: Liver injury), Various SNPs (3: Efficacy in multiple sclerosis)	1) HLA-DRB1*04, HLA-DRB1*15, rs9272105, rs4961252 (Neutralizing antibody)^42,72^	Limited data on metabolism/transporter pathways	Liver injury, depression, heart failure, leukopenia, flu-like symptoms, and etc.
Tocilizumab	33	−/−	*IL6R, CD69, FCGR3A, GALNT18* (3: Efficacy in rheumatoid arthritis)	–	Limited data on metabolism/transporter pathways	Thrombocytopenia, liver injury, neutropenia, rash, hypertension, infection
Ruxolitinib	13	−/−	–	–	Inactivation mainly via CYP3A4 and partly via CYP2C9	Cytopenia, dyslipidemia, infection, liver injury
Baricitinib	11	−/−	–	–	Efflux transport mainly via OAT3 and partly via BCRP and p-glycoprotein. Inactivation partly via CYP3A4.	Thrombosis, infection, gastrointestinal perforation, cytopenia, dyslipidemia, liver injury
Corticosteroids	23	−/−	Various SNPs (3: Efficacy/toxicity of various conditions)	Various SNPs (Efficacy/toxicity of various conditions)^53^	Inactivation mainly via CYP3A4 and CYP3A5. Efflux transport via P-glycoprotein.	Infection, psychosis, gastritis, glucose fluctuations, osteonecrosis, etc.

*COVID-19* coronavirus disease 2019, *CV* cardiovascular, *FDA* the U.S. Food and Drug Administration, *GI* gastrointestinal, *HCQ* hydroxychloroquine, *HIV* human Immunodeficiency virus, *IL* interleukin, *N/A* not applicable, *PK* pharmacokinetics, *SNP* single nucleotide polymorphism, *CNT* concentrative nucleoside transporter.

^a^The number of interventional trials for COVID-19 registered in ClinicalTrials.gov as of 6/4/2020.

^b^Information listed on Table of Pharmacogenomic Biomarkers in Drug Labeling and Table of Pharmacogenetic Associations on FDA website accessed on April 21, 2020.

^c^Only included plausible genes involving the index drug when gene-effect data was derived from a multiple drug regimen. Levels of evidence in PharmGKB: 1 = high; 2 = moderate; 3 = low; 4 = preliminary.

^d^N/A indicates that level of evidence is not available. Gene-effect pairs are reported in PharmGKB Variant Annotations.

Dash (–) indicates no data available.

### Hydroxychloroquine and chloroquine

The first antiviral drugs officially indicated for COVID-19 in the U.S. were hydroxychloroquine and chloroquine as an FDA Emergency Use Authorization issued on March 28, 2020^8^. This was later revoked on June 15, 2020 based on new data suggesting that the drug’s potential benefits may not outweigh its known and potential risks^9^. However, a number of ongoing studies for both treatment and prevention of COVID-19 are still underway. Hydroxychloroquine sulfate and chloroquine phosphate have been used for malaria and autoimmune disorders for decades. It is thought to increase pH of phagolysosome and, thereby, interrupts virus fusion, and it also prevents binding of the virus to cell surface receptors^10^. The immunomodulatory effects of these drugs may also play a role in managing the cytokine storm associated with advanced COVID-19 disease. Concerns around the heart rhythm problems of these agents for treatment of COVID-19 has been announced from the FDA^11^. This is corroborated by a large retrospective study of hospitalized patients with COVID-19 in New York metropolitan region; those treated with hydroxychloroquine and azithromycin had a higher incidence of cardiac arrest and possible higher mortality^12^. The NIH guidelines currently do not recommend the use of hydroxychloroquine or chloroquine^3^. Although the role of these drugs in COVID-19 may be limited, biomarkers for toxicity may potentially be valuable. Hydroxychloroquine and chloroquine are metabolized via cytochrome P450 (CYP) enzymes including CYP2C8, CYP3A4, and CYP2D6^13^. These drugs are substrates of organic anion transporting polypeptides (OATP), influx cellular membrane transporters encoded by *SLCO*^14^. The genes involved in the pharmacokinetics of hydroxychloroquine and chloroquine are considered “very important pharmacogenes (VIPs)” by the PharmGKB, genes that are particularly important in the field of pharmacogenomics.

A study in 194 patients with systemic lupus erythematosus showed that metabolism ratio of the active metabolite of hydroxychloroquine to its parent drug was increased by ~20% in those carrying variants in *CYP2D6* (rs1135840: CC vs. GG was 0.90 vs. 0.69, respectively, *p* < 0.01)^15^. In a cohort of 164 malaria-infected patients, low-activity alleles of *CYP2C8* (i.e., *2, *3, and *4) were associated with worse reduction of gametocytemia than wild-type alleles 1 day after chloroquine/primaquine treatment (−2.21 vs. −11.18 gametocytes/μL, *p* = 0.007, respectively)^16^. In the same cohort, a subsequent analysis showed that variant alleles of *SLCO1A2* and *SLCO1B1* had lower gametocytemia clearance than the wild-type alleles after adjusting for *CYP2C8* (*p* = 0.018 and 0.024, respectively)^14^.

Hydroxychloroquine and chloroquine are relatively safe drugs when used for malaria and autoimmune disease, although there are several toxicities of importance. Individuals who are glucose-6-phosphate dehydrogenase (G6PD) deficient are considered at higher risk of hemolytic anemia upon exposure to hydroxychloroquine or chloroquine. However this has not been consistently shown. No individuals developed hemolytic anemia among a group of 74 patients with G6PD deficiency who received a 3-day course of chloroquine with methylene blue, nor another group of 11 patients who received a total cumulative hydroxychloroquine exposure of 700 person-months^17,18^. Another well-recognized adverse effect of hydroxychloroquine and chloroquine is retinopathy, which occurs more frequently with long term use (in years) and higher doses. Retinopathy occurred less frequently in those with the minor allele of *ABCA4* c.5814A>G (OR: 0.01, 95% CI: 0.00–0.27, adjusted for clinical factors including treatment duration and dose)^19^. Although data on the functional analysis of this variant is limited, mutations in *ABCA4* are associated with various retinal diseases such as Stargardt disease, which is phenotypically similar to chloroquine-induced retinopathy^20^. The average treatment duration in the study was approximately 11 years, thus the effects of *ABCA4* c.5814A>G in short-term use of hydroxychloroquine or chloroquine for COVID-19 treatment would likely to be small. Of note, hydroxychloroquine is also a CYP2D6 inhibitor, which may reduce CYP2D6 activity. This is particularly of concern when other CYP2D6 substrates that also prolong QT interval, such as ondansetron and haloperidol, are used concomitantly with hydroxychloroquine and chloroquine since these combinations may significantly reduce their metabolism which may further potentiate QT prolongation.

### Azithromycin

Azithromycin is a macrolide antibacterial agent with anti-inflammatory properties. A recent observational study showed that azithromycin, when used in combination with hydroxychloroquine, may be more effective than hydroxychloroquine alone for COVID-19^21^. Although macrolide agents are associated with numerous drug–drug interactions, azithromycin is not a significant substrate of CYP3A4, SLCO1B1, or SLCO1B3, and therefore has fewer interactions than erythromycin or clarithromycin^22^. The pharmacokinetics of azithromycin are, however, influenced by the activity of P-glycoprotein transporter encoded by *ABCB1*. Genetic variation in *ABCB1* showed up to a 2-fold lower peak azithromycin concentrations in 20 healthy volunteers after a single dose (rs2032582TT/rs1045642TT vs. rs2032582GG/rs1045642CC was 468.0 vs. 911.2 ng/ml, respectively, *p* = 0.013)^23^. Higher systemic exposure to azithromycin is of particular concern when it is combined with hydroxychloroquine or chloroquine because of their additive effects on QT prolongation which may result in fatal arrhythmias.

### RNA polymerase inhibitors (remdesivir, ribavirin, and favipiravir)

Nucleotide analogs, including remdesivir, ribavirin and favipiravir, inhibit viral RNA polymerase after metabolism to their active forms by intracellular enzymes. Remdesivir became available for severe COVID-19 in the U.S. via FDA Emergency Use Authorization on May 1, 2020^24^, following two randomized, double-blinded, placebo-controlled trials suggesting potential benefits outweighing the known and potential risks^25,26^. Although no pharmacogenomic data of remdesivir are available, in vitro studies suggest that it is a substrate for drug metabolizing enzymes CYP2C8, CYP2D6, and CYP3A4, and is a substrate for OATP1B1 and P-glycoprotein transporters^27^. Thus, known variants of these genes could theoretically affect the pharmacokinetics of remdesivir^28–30^. All of these genes are considered VIPs by PharmGKB.

Ribavirin is indicated for hepatitis C virus infection and is also under investigation for COVID-19. Genetic polymorphisms in influx cellular transporters of ribavirin result in up to 30% variability in the trough concentration; troughs were significantly higher in those with *SLC29A1* variants (homozygous for the variants 2070 ng/ml vs. wild-type 1837 ng/ml; *p* = 0.02), while significantly lower in those with *SLC28A2* variants (homozygous for the variants 1595 ng/ml vs. heterozygous 1933 ng/ml vs. homozygous wild-type 2229 ng/ml; *p* = 0.04) and *SLC28A3* variants (homozygous 2294 ng/ml vs. heterozygous 1813 ng/ml; *p* = 0.01)^31^. It is well-recognized that various *ITPA* (inosine triphosphatase) variants have protective effects against hemolytic anemia, which is the most common and dose-limiting adverse effect of ribavirin^32^. Decreased ITPA activity in red blood cells leads to accumulation of inosine triphosphate and protects against ribavirin-induced hemolysis. In a meta-analysis of 20 studies, hemoglobin decline was associated with wild-type alleles of *ITPA*; rs1127354 CC (OR: 12.8, 95% CI: 7.4–22.1), rs7270101 AA (OR: 3.4, 95% CI: 2.1–5.6), and rs6051702 AA (OR: 4.4, 95% CI: 2.8–7.0)^33^. A model incorporating *ITPA* genotypes and clinical factors was predictive of the degree of ribavirin-related hemoglobin decrease^34^. Of note, hemolytic anemia was also reported from a short-term use of ribavirin for respiratory virus infection^35^. In contrast, the *ITPA* variants in rs6139030 were identified to be a risk of thrombocytopenia in a genome-wide association study among 303 patients with hepatitis C who received ribavirin and peg-interferon (OR: 3.9, 95% CI: 2.8–5.5, *p* = 1.33 ×10^–15^)^36^.

Favipiravir was developed and approved in Japan in 2014 exclusively for a resistant, novel influenza pandemic and is now under investigation for COVID-19. Although no published studies specifically have addressed its pharmacogenomics, it is metabolized by aldehyde oxidase and partly via xanthine oxidase^37^. Notably, variants of aldehyde oxidase are associated with pharmacodynamic outcomes in other drugs which are substrates of aldehyde oxidase such as azathioprine or allopurinol^38^.

### Anti-retrovirus agents (lopinavir/ritonavir, darunavir/cobicistat)

Lopinavir is a viral protease inhibitor that is primarily used in the treatment of human immunodeficiency virus (HIV), but it has also been used in COVID-19. HIV-protease inhibitors including lopinavir/ritonavir are currently not recommended for COVID-19 because of lack of efficacy in a small randomized controlled trial and a concern of inadequate drug exposure relative to that required for SARS-CoV-2 inhibition^3^. Ritonavir inhibits the inactivation of lopinavir via the CYP3A4 pathway and boosts the concentrations of lopinavir and is marketed as a combination product. Besides CYP3A, several pathways are involved in the pharmacokinetics of lopinavir, including other CYP enzymes and membrane drug transporters. A pharmacogenomic analysis of lopinavir/ritonavir, including 1380 variants in 638 HIV-infected Caucasians, identified four significant variants. Clearance of lopinavir in a population PK model was higher in individuals with SLCO1B1*4/*4 and lower in individuals with two or more variant alleles of SLCO1B1*5, *ABCC2* or a *CYP3A* tag compared to the reference group (12.6 vs. 3.9 vs. 5.4 l/h, respectively, *p* < 0.01)^39^. Another genetic association study explored 290 variants for their effects on lopinavir/ritonavir related toxicity among 104 Caucasian patients with HIV; variants in the *CETP*, *MCP‑1*, *ABCC2*, *LEP*, and *SLCO1B3* genes were associated with dyslipidemia and hyperbilirubinemia, and a variant in *IL-6* was associated with diarrhea (all *p* < 0.01)^40^.

Darunavir, also a protease inhibitor used in HIV treatment, is a substrate of CYP3A4 that is used simultaneously with a CYP3A4 inhibitor, cobicistat, in a clinical trial for COVID-19^2^. Several haplotypes in *CYP3A4* have been discovered that affect the activity and/or expression of CYP3A4^30^. Those haplotypes could hypothetically affect concentrations of these drugs, but they have yet to be studied specifically with darunavir. Although there is no direct evidence that darunavir is a substrate for SLCO3A1, a 12% significantly lower darunavir clearance was observed in carriers of an *SLCO3A1* variant (*p* < 0.05)^41^.

### Interferon (INF) β-1b

A family of IFNs, particularly IFN-β1b, has showed efficacy against SARS- and/or MERS-coronaviruses and are currently being investigated for COVID-19 either alone or in combination with other therapy (e.g., lopinavir/ritonavir)^2^. As it is commonly seen in other biologic drugs, pharmacogenomics determinants are not well-delineated for IFN-β1b. In contrast, reduced efficacy and increased adverse effects secondary to immunogenicity is a specific concern among biologics. In a cohort of Swedish patients with multiple sclerosis who received IFN-β1b, the risk of biologically relevant neutralizing antibody development was higher in patients with the HLA-DRB1*04 allele (OR: 3.53, 95% CI: 1.64–7.61) and lower with HLA-DRB1*15 (OR: 0.33, 95% CI: 0.16–0.71)^42^. In a two-stage genome-wide association study among 56 cases and 126 controls of IFN-β1b-treated patients with multiple sclerosis, a higher risk of drug-induced liver injury was identified (*p* = 2.3 × 10^−8^, OR: 8.3, 95% CI: 3.6–19.2) in patients with variants of *IRF6*, which encodes for an interferon regulatory factor and is involved in promotion of liver damage^43^. The results were confirmed in an independent cohort of multiple sclerosis patients for an association with increased peak levels of aspartate aminotransferase (*p* = 7.6 × 10^−5^) and alkaline phosphatase (*p* = 4.9 × 10^−4^)^43^.

### IL-6 and IL-1 antagonists (tocilizumab, sarilumab, siltuximab, anakinra)

Severe COVID-19 is associated with a cytokine-release syndrome with elevated interleukin-6 (IL-6)^44^. Tocilizumab, an inhibitor of the IL-6 receptor, is commonly used for rheumatoid arthritis (RA) and cytokine-release syndrome induced by chimeric antigen receptor-T cell therapy, and now it is under investigation for COVID-19. Although several genetic biomarkers have been reported in the efficacy of tocilizumab in RA, including *FCGR3A, IL6R, CD69, GALNT18*^45–47^, potential translation of these data to COVID-19 is highly speculative. No studies have addressed pharmacogenomics of tocilizumab in patients with cytokine-release syndrome, which is similar to the physiology in COVID-19. The only genetic variants potentially involving tocilizumab’s pharmacokinetics are in the *FCGR3A* gene, where differences in efficacy is postulated to be due to changes in systemic exposure. In 87 patients with RA treated with tocilizumab, *FCGR3A* rs396991TT genotype showed higher response at 12 months (vs. GT; OR: 5.1; 95% CI: 1.2–21.3; *p* = 0.03). This variant may affect the affinity of the Fc fragment of IgG receptor to tocilizumab and alter its systemic clearance^45^. Polymorphisms of *IL6R* are considered to affect intracellular signaling pathway of IL-6 receptor bound to tocilizumab, which may also be applicable to other conditions with upregulated IL-6 pathway^46^. In contrast, variants in *CD69* and *GALNT18* are thought to have limited direct effects on tocilizumab. Variants in those genes are more likely to affect the downstream signaling pathways of the immune system in RA patients, which may limit generalization to non-RA patients^47^. At this point there is limited evidence that pharmacogenomic biomarkers would be helpful in determining response to tocilizumab therapy in COVID-19. No relevant pharmacogenomic data is reported in other IL-6 or IL-1 antagonists (i.e., sarilumab, siltuximab, anakinra).

### Janus kinase inhibitors (ruxolitinib, baricitinib)

A group of Janus kinase inhibitors is another potentially effective immunomodulator for COVID-19 that is currently in clinical trials. Ruxolitinib is approved by the FDA for myeloproliferative diseases and graft-versus-host-disease, and baricitinib for RA. No published studies have addressed the effects of genetic variants on either of the two drugs in any patient population. However, their pharmacokinetics pathways involve a few potentially important pharmacogenes. Ruxolitinib is a major and baricitinib is a minor substrate of CYP3A4^48,49^. Ruxolitinib is also partly metabolized by CYP2C9. Both of these genes are considered VIPs in the PharmGKB and subject to notable genetic polymorphisms^30,50^. Although the main pharmacogene of baricitinib, *SLC22A8* encoding OAT3 transporter^49^, is not a VIP, influence of variants on its activity is previously reported in another substrate drug^51^.

### Corticosteroids

The potential role of corticosteroids in the treatment of COVID-19-infected patients is mainly limited to those with acute respiratory distress syndrome (ARDS). In patients with SARS-coronavirus infection, corticosteroid use was associated with delayed viral clearance but also showed possible benefits in those with ARDS^52^. Many variants have been associated with corticosteroids response and toxicities across multiple disease conditions, including genes involved in the receptor binding (e.g., *CRHR1*, *NR3C1*), chaperone/cochaperone protein (e.g., *ST13*, *STIP1*, *FKBP5*), metabolizing enzymes (e.g., *CYP3A4*, *CYP3A5*, *CYP3A7*, *GSTT1*), and transporters (e.g., *MDR1*, *ABCB1*)^53^. The mechanistic and metabolic pathways of steroids are complex, and genomic determinants with sufficient evidence for clinical application to COVID-19 were not identified. Variants associated with corticosteroids in PharmGKB with level of evidence higher than “low” (level 3) were only assessed in combination therapy (e.g., with chemotherapy) or inhaled corticosteroids. No pharmacogenetic information specifically on the effectiveness of corticosteroids for ARDS was found.

### Summary of clinical implications of pharmacogenomics for COVID-19

We found evidence that several genetic variants may alter the pharmacokinetics of hydroxychloroquine, azithromycin, ribavirin, lopinavir/ritonavir and possibly tocilizumab, which hypothetically may affect clinical response and toxicity in the treatment of COVID-19. Although the level of evidence for most is weak, and has not been directly studied in patients with COVID-19, some of these potential pharmacogenetic associations are worth further exploration.

QT prolongation could hypothetically be exacerbated by a combination of drug–drug, drug–gene, and drug–disease interactions in the treatment of COVID-19. As previously described in this review, hydroxychloroquine, chloroquine and azithromycin can individually increase risk for QT prolongation, and those drugs have been used in combination in COVID-19 patients. This combination therapy showed higher odds of cardiac arrest in hospitalized patients with COVID-19 in comparison to those on neither therapy (odds ratio 2.13 [95% CI: 1.12–4.05])^12^. Hydroxychloroquine is a CYP2D6 inhibitor, which calls for a vigilance in drug–drug interactions in patients with COVID-19, who are at risk of polypharmacy. In particular, some QT-prolonging drugs, that are commonly used in hospitalized patients, are substrates of CYP2D6 which may be inhibited by hydroxychloroquine. This may dramatically increase the risk of QT prolongation. The CYP2D6*4 nonfunctional allele is present in nearly 20% of patients with European ancestry^54^, and thus patients with the CYP2D6*4 allele may have even higher risk. Patients with the *ABCB1* 2677GG/3435CC genotype have higher peak concentrations of azithromycin, a QT-prolonging drug^23^. Higher concentrations of azithromycin in combination with hydroxychloroquine may be particularly dangerous. Baseline QT interval evaluation prior to therapy is important since there is a greater risk of prolongation in individuals with high baseline values. The prevalence of high baseline QT intervals was reported to be 14.5% among those aged ≥40 years in a US population study^55^. And finally, the potential combination of these arrhythmogenic risks may be further exacerbated in the setting of COVID-19, in which the disease is associated with myocardial injury and arrhythmias^56^.

The association between IFN-β1b and a variant in *IRF6* for the adverse outcome of liver damage was one of the strongest pharmacogenetic associations identified in this review^43^. The previous studies of IFN-β1b/*IRF6* were performed in patients with multiple sclerosis, not COVID-19. Therefore the IFN-β1b/*IRF6* pharmacogenetic association should be investigated in patients with COVID-19, especially since a large portion of patients with COVID-19 have abnormal liver function tests and liver injury^57^. Individuals homozygous for SLCO1B1*4 showed a greater than 2-fold increase in lopinavir clearance. Notably, this variant has a differing allele frequency across racial groups; it is 14% in European, 6% in African, and 0.3% in East Asian descents. Although G6PD deficiency is associated with hydroxychloroquine and chloroquine related hemolytic anemia, the evidence suggests that the strength of the association is unclear and possibly because there are varying degrees of G6PD deficiency conferred by the genetic variations. The *ITPA* variants, associated with protective effects against ribavirin-related hemolytic anemia, has PharmGKB level 2 evidence (moderate strength), may have a role in improving ribarivin safety.

Older age, race, male gender, obesity and comorbid conditions have been established as risk factors for COVID-19 susceptibility and death^58,59^. Whether they are also precise risk factors for drug treatment failure have yet to be established. It has yet to be determined if host genomics or confounding factors (e.g., access to healthcare, environment) also play a role. Several studies though suggest that higher susceptibility to COVID-19 is associated with genetic variants, including those in the *ACE1*, *ACE2*, *TMPRSS2*, *GSTT1* genes^60–62^. Interestingly, several important *ACE2* variants on the X-linked locus were identified in male as hemizygous, which may explain the higher mortality in male^63^.

## Conclusion

Although no direct evidence of pharmacogenomics data in patients with COVID-19 was available at this time, there are plausible mechanisms by which genetic determinants may play a role. Studies must be conducted in COVID-19 before pharmacogenomic testing can be recommended. These data support the collection of DNA samples for pharmacogenomic studies of the hundreds of currently ongoing clinical trials of COVID-19 therapies. One of the biggest success stories in the field of pharmacogenomics was for a drug used to treat another, highly lethal, infectious disease: abacavir for HIV. The application of a pharmacogenetic test (HLA-B*57:01) nearly eliminates a potentially fatal hypersensitivity reaction to abacavir^64^. Thus, the pharmacogenetic test for abacavir is now standard of care in the treatment of HIV. It took years after the discovery of HIV to develop such a pharmacogenetic success for abacavir. While it is understandable, at these very early stages since the discovery of COVID-19, the pharmacogenetic data are very limited. The use of modern genomic tools coupled with a proactive assessment of the most likely gene-drug candidates could lead to a quicker understanding of the role of pharmacogenetics for COVID-19. Important in silico efforts are underway to repurpose old drugs for COVID-19 and some of these drugs may have established pharmacogenomic markers in other disease^65,66^.

It is worth noting potential limitations of the use of pharmacogenetics in the treatment of COVID-19. In an acute illness such as COVID-19, pharmacogenetics would only be useful if the genetic test results were already available (i.e., pre-emptive pharmacogenetic testing) or rapidly available (i.e., point-of-care genetic testing). Several institutions have already implemented pre-emptive pharmacogenetic testing, and some patients may have results readily available^67^. Point-of-care pharmacogenetic tests are available but generally the potential variants of relevance in COVID-19 therapies are not available on this type of testing platform^68^.

In the face of unprecedented challenges posed by the COVID-19 pandemic, collaborative efforts among the medical communities are more important than ever to improve the efficacy of these treatments and ensure safety. Some large national COVID-19 trials are evaluating pharmacogenomics, which will inform the role of pharmacogenomics markers for future clinical use.

## Data Availability

No datasets were generated or analyzed during the current study.
